# Susceptibility of neuron-like cells derived from bovine Wharton’s jelly to bovine *herpesvirus* type 5 infections

**DOI:** 10.1186/1746-6148-8-242

**Published:** 2012-12-10

**Authors:** Tereza C Cardoso, Juliana B Novais, Talita F Antello, Camila Silva-Frade, Marina C Ferrarezi, Heitor F Ferrari, Roberto Gameiro, Eduardo F Flores

**Affiliations:** 1 UNESP – University of São Paulo State, Laboratory of Animal Virology and Cell Culture, São Paulo, Brazil; 2 UNESP – University of São Paulo State, Embryology Laboratory, Faculty of Veterinary Medicine, São Paulo, Araçatuba, 16050-680, Brazil; 3 UNESP – University of São Paulo State, Federal University of Santa Maria, Virology Section, Santa Maria, RS, 97115-900, Brazil

**Keywords:** BoHV-5, *in vitro* replication, Neuronal culture

## Abstract

**Background:**

Bovine herpesvirus type 5 (BoHV-5), frequently lethal in cattle, is associated with significant agricultural economic losses due to neurological disease. Cattle and rabbits are frequently used as models to study the biology and pathogenesis of BoHV-5 infection. In particular, neural invasion and proliferation are two of the factors important in BoHV-5 infection. The present study investigated the potential of bovine Wharton’s jelly mesenchymal stromal cells (bWJ-MSCs) to differentiate into a neuronal phenotype and support robust BoHV-5 replication.

**Results:**

Upon inducing differentiation within a defined neuronal specific medium, most bWJ-MSCs acquired the distinctive neuronal morphological features and stained positively for the neuronal/glial markers MAP2 (neuronal microtubule associated protein 2), N200 (neurofilament 200), NT3 (neutrophin 3), tau and GFAP (glial fibrillary acidic protein). Expression of nestin, N200, β-tubulin III (TuJI) and GFAP was further demonstrated by reverse transcriptase polymerase chain reaction (RT-PCR). Following BoHV-5 inoculation, there were low rates of cell detachment, good cell viability at 96 h post-infection (p.i.), and small vesicles developed along neuronal branches. Levels of BoHV-5 antigens and DNA were associated with the peak in viral titres at 72 h p.i. BoHV-5 glycoprotein C mRNA expression was significantly correlated with production of progeny virus at 72 h p.i. (p < 0.05).

**Conclusion:**

The results demonstrated the ability of bWJ-MSCs to differentiate into a neuronal phenotype *in vitro* and support productive BoHV-5 replication. These findings constitute a remarkable contribution to the *in vitro* study of neurotropic viruses. This work may pave the way for bWJ-MSCs to be used as an alternative to animal models in the study of BoHV-5 biology.

## Background

The two most basic properties of stem cells are their capacity for indefinite self-renewal and differentiation into multiple cell or tissue types [1-3]. Embryonic stem cells are totipotent and can be maintained in culture in the presence of leukemia inhibitory factor (LIF) [4]. Withdrawal of LIF induces the formation of cellular aggregates called embryonic bodies. A wide variety of cell types migrate away from embryonic bodies, including some with neuron-like morphology [5]. In addition, embryonic stem cells may be differentiated into neurons and glial cells with retinoic acid or basic fibroblast growth factor (bFGF) [6,7].

Neurons derived from embryonic stem cells express neurofilaments, neuron-specific class III β-tubulin (TuJl) and a number of neuron-specific microtubule-associated proteins [8]. Neural stem cells (NSC) are immature, uncommitted cells that exist in both the developing brain and the adult nervous system [8]. These cells can undergo expansion and differentiate into neurons, astrocytes, and oligodendrocytes [8,9]. Stem cells differentiated into neurons from both these sources display a limited proliferation potential. Obtaining them requires invasive procedures and results in ethical limitations in their use [10]. Because neuroblastoma cells can mimic the morphological and biochemical characteristics of primary neurons, they have proved to be highly valuable models in analyzing the neuropathogenesis and neurotropism of a range of viruses [11]. To overcome ethical issues regarding the use of fetal and adult brain samples, mesenchymal cells (MSCs) isolated from the Wharton’s jelly (WJ) of human and animal umbilical cords (UC) represent an attractive alternative. These cells are easily obtained, proliferate rapidly in culture, are immunologically compatible, and represent fetal adnexa that is usually discarded [1,5]. MSCs have been previously isolated from dogs, sheep, horses [2,3,12], and recently bovine WJ-UC [13]. The WJ-UC cell structure is embryonic in origin and encloses the yolk sac, which is the source of the primordial germ cells and the first hematopoietic stem cells [5].

The neurotropic RNA and DNA viruses that produce central nervous system (CNS) diseases employ a range of pathologic mechanisms in a variety of hosts. The useful biological models applied to isolate, propagate, and study biological properties of these viruses include mice, rabbits, monkeys and hamsters. The α-herpesviruses, including human herpes simplex types 1 and 2 (HSV-1 and HSV-2), varicella-zoster virus (VZV), animal pathogens such as pseudorabies virus (PRV), bovine herpesvirus types 1 and 5 (BoHV-1 and BoHV-5), and Marek’s disease virus are neurotropic viruses capable of invading the peripheral and central nervous systems to cause neurological disease [14]. BoHV-5 belongs to the family *Herpesviridae*, subfamily α-*Herpesvirinae*, and genus *Varicellovirus*. BoHV-5 is genetically and antigenically closely related to BoHV-1, a highly prevalent virus responsible for respiratory and genital disease in cattle around the world [15].

BoHV-1, associated with respiratory and genital disease, and BoHV-5, associated with neurological disease, cause significant agricultural loss. [16]. Experimentally infected cattle and/or rabbits have been used to better understand the neural invasion and spread of BoHV-1 and BoHV-5 [16-19]. Indeed, rabbits have been used as an animal model to study selected aspects of acute and latent infections caused by BoHV-5 [19]. Unlike BoHV-1, BoHV-5 can be difficult to isolate from cell culture and animals presenting neurological symptoms after experimental inoculation [20-23]. These difficulties in viral isolation might be related to low virus titres present in tissue sections [18].

In contrast with *in vivo* studies, *in vitro* studies with Madin-Darby bovine kidney (MDBK) cells and primary cell cultures of bovine origin represent adequate substrates for viral *in vitro* studies [24,25]. In cell culture, BoHV-1 and BoHV-5 share similar phenotypes and produce similar cytopathic effects (CPE). CPE are characterized by a round cell shape and presence of multinucleated and syncytial cells [25]. In a previous report, the CPE observed in bovine lung cells infected with BoHV-1 were elongated and spindle-shape cells; in contrast, BoHV-5 infected cells showed syncytial-like CPE [26]. In spite of many reports describing the difficulty of re-isolating the virus from field samples and experimentally infected rabbits, the MDBK cell line has been used as a standard substrate for BoHV-5 isolation and propagation *in vitro*[25]. To date, no effort has been made to develop a better *in vitro* culture system for virus isolation/propagation. Optimizing viral culture would reduce the need for the use of animal models. In this context, the aim of the present study was to generate a novel *in vitro* biological model/substrate that would differentiate bovine WJ-MSC cells into neuron-like cells susceptible to neurotropic BoHV-5 replication. In addition, such a model would provide a means for rapidly examining key aspects of the interactions between BoHV-5 and bovine neuron-like cells. In particular, this model system may overcome many of the limitations of live animals, primary neuronal culture, or neural mesenchymal cells from fetuses with less costly experimentation and ethical constraints.

## Results

### Differentiation of stem cells into neuron-like cells

**Figure 1:**
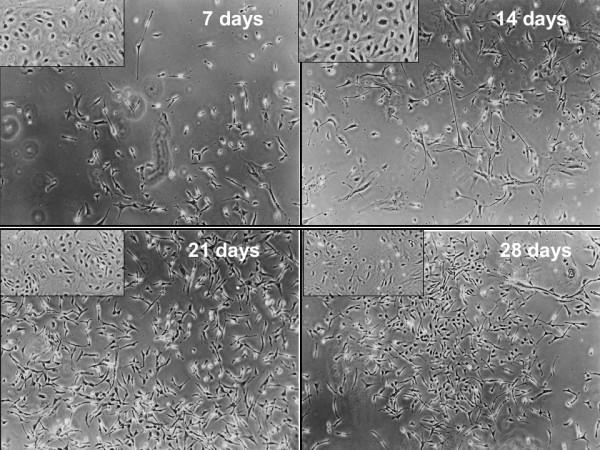
**Bovine Wharton’s jelly differentiation.** Photomicrographs representative of the morphological appearance of neuron-like cells under differentiation at 7, 14, 21 and 28 days of neuron induction observed under phase contrast microscopy (x 40 magnification). The multipolar, round cell bodies form a network-like structure. The inset images show the undifferentiated bWJ-MSCs.

**Figure 2:**
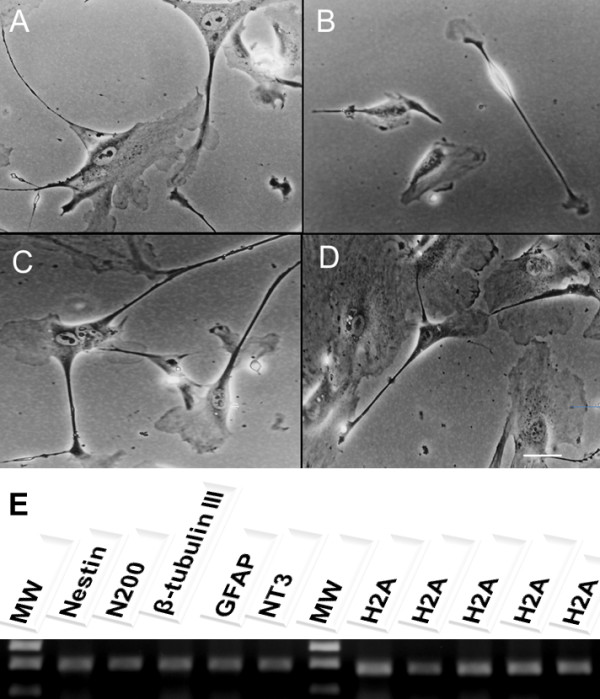
**Neuron-like cells characterization.** After 28 days of neurogenic induction, a neuron-like morphology was visible under phase contrast microscopy. **A**) Neuron-like cells with triangular, round and cone-shaped morphology; **B**) Bi-polar neuron-like morphology; **C**) Network among neuron-like cells; **D**) Long axon of a Golgi neuron;x 100 magnification; **E**) RT-PCR analysis of neuronal specific markers:nestin, N200, β-tubulin III, GFAP and NT3 transcript expression in neuron-like cells after differentiation were observed. The H2A gene was used as an internal control for the reverse transcription-polymerase chain reaction. MW-molecular weight 1 kb plus.

**Figure 3:**
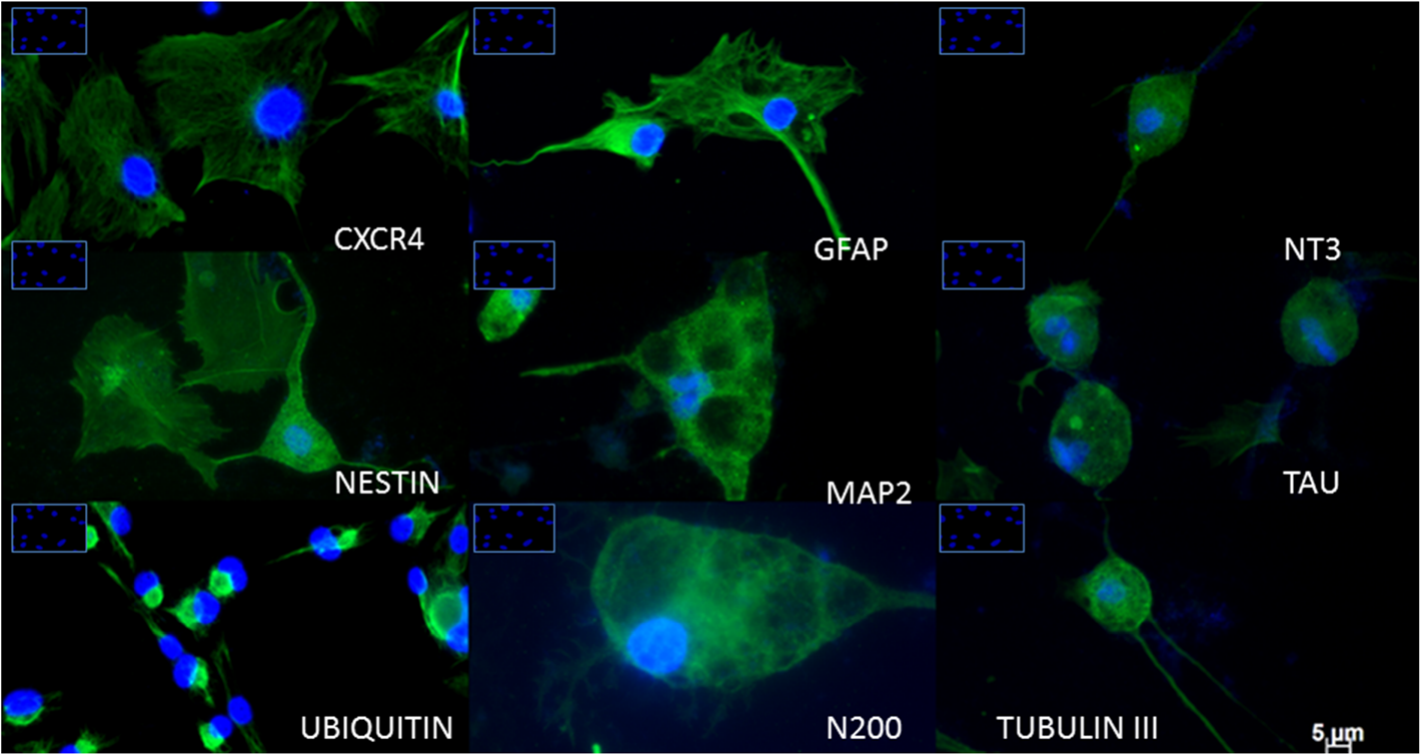
**Neuron-like cells surface markers.** Immunofluorescence images showing the expression of neuronal markers, such as CXCR4, GFAP, NT3, nestin, MAP2, Tau, ubiquitin, N200, and TuJI, by immunofluorescence staining. DAPI indicates the nucleus. Inset images show the absence of neuronal markers in the undifferentiated bWJ-MSCs (scale bar: 20 μm).

The main objective of the present study was to assess the full neural potential of bWJ-MSCs after culture in a retinoic acid-deprived reprogramming medium. Continuous culture for 28 days resulted in cells exhibiting varied morphologies, including cells with multiple long thin-axon-like processes at 7, 14, 21 and 28 days (Figure 1A-D). Furthermore, we observed the presence of neurons and oligodendrocytes based on morphology of spindle-like cells with multiple, network forming (Figure 2C and 2D), long-branch projections (Figure 2A). In addition, neural-like cells displayed distinct neuronal morphology, ranging from simple bipolar to large, extensively branched, multipolar cells (Figure 3). Thus, after 28 days of neuron induction, bWJ-Uc cells were capable of generating both neuron and glial-like cells.

### Characterization of differentiated neuron-like cells

**Figure 4:**
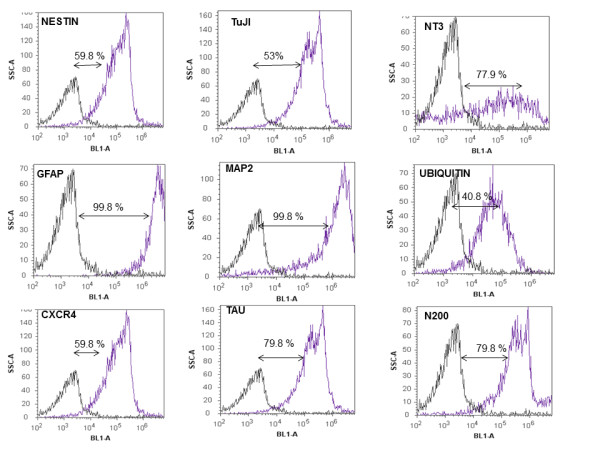
**Neuron-like cells phenotype.** Flow cytometric analysis of surface marker expression after neuronal induction of bovine-derived UC-WJ cells. The data from the negative isotype control and the respective surface markers for mesenchymal stromal cells are represented by the histogram. The data shown are representative of the cell phenotype after neuronal induction. The data were processed using an Attune™ acoustic focusing cytometer, and auto-fluorescence was excluded as a global compensation tool (≤ 10^3^). The y-axis represents a log scale.

The distribution of neural/glial cell markers in differentiated cells is shown in Table 1. Immunostaining for neuro-differentiation markers displayed 75-100% expression of N200, MAP2, NT3, Tau, and GFAP. Approximately 50-75% of differentiated cells were positive for nestin, CXCR4, and TuJI. The ubiquitin marker was less frequently detected among differentiated cells (25-50%). Consistent with these observations, RT-PCR confirmed presence of nestin, N200, β-tubulin III (TuJI), GFAP and NT3 mRNA (Figure 2E). Flow cytometric analysis was used to determine the phenotype of the neuron-like cells. Cell-surface markers that characterize neuro-differentiation at 28 days of culture were analyzed. The results showed that cells expressed at least 70% of putative neuronal markers such as NT3, GFAP, MAP2, Tau and N200 (Figure 4). Nestin, β-tubulin III, ubiquitin, and CXCR4 were also expressed, but at lower rates (Figure 4). Our experiments confirmed previous studies demonstrating the ability of human MSCs derived from Wharton’s jelly layer to generate neuron-like cells. Our results also demonstrated cross-reactivity among human and bovine monoclonal antibodies against these markers.

**Table 1:** Incidence of various markers that neuron-like cells derived from bWJ-UC express after 28 days of differentiation visually counting under UV microscopy

**Marker**	**Neuron-like cells**	**bWJ-UC**
Nestin	+++^a^	_
MAP2	++++	_
GFAP	++++	_
N200	++++	+
NT3	++++	_
TuJI	+++	_
Tau	++++	_
Ubiquitin	++	++
CXCR4	+++	_
Bovine IgG	_	_
Bovine IgM	_	_

^a^Values were determined by visually counting positive cells (−, 0%; +, 0-25%; ++, 25-50%; +++, 50-75%; ++++, 75-100%).

### Susceptibility of neuron-like cells to BoHV-5 infection

**Figure 5:**
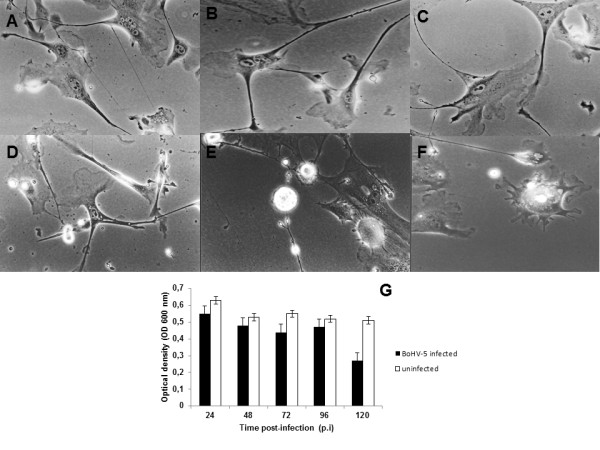
**Bovine *Herpesvirus* type 5 infection.** Neuron-like cells infected with BoHV-5 demonstrating cytopathic effects **A**) at 24 h post-infection (p.i.) showing initial enlargement of cell branches; and **B** and **C**) Vesicles forming between networks at 48 h p.i.; **D-F**) Visible vesicles along cell branches, cell bodies and close to networks at 72 to 120 h p.i.; **G**) Viability of neuron-like cells was measured with a MTT-based assay in BoHV-5-infected and -uninfected cells at different times p.i. The data are expressed as the mean ± standard deviation (s.d.).

**Figure 6:**
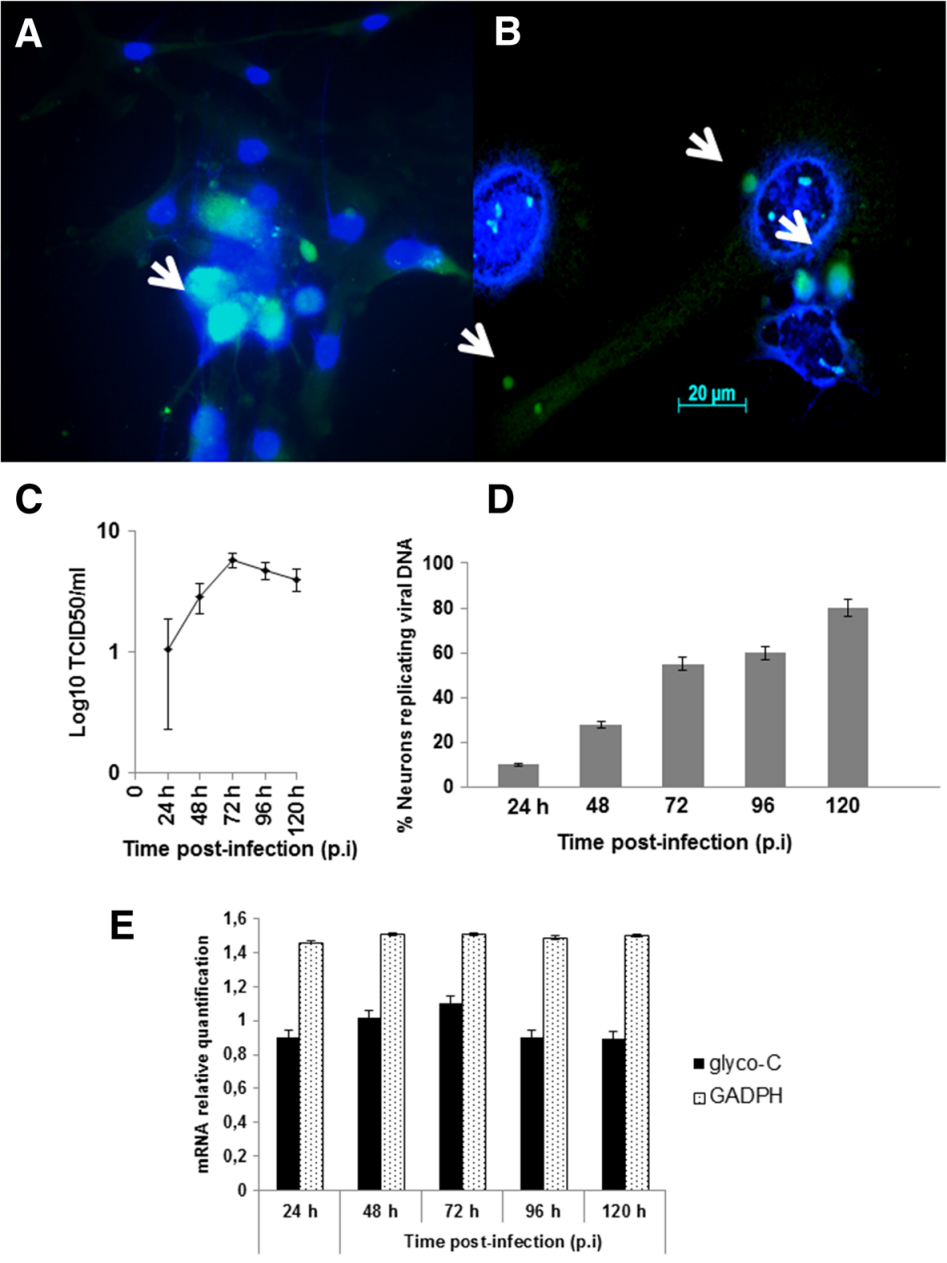
**Identification of BoHV-5 infection.** Detection of BoHV-5 antigens, viral DNA and *glyco*-*C* m RNA expression in infected bWJ-MSCs differentiated into neuron-like cells. **A**) Intense fluorescence positive signal for BoHV-5 *glyco*-C protein at 72 h post-infection (arrow head); **B**) Positive grains observed in the neuron-like cell’s nucleus, outside the nuclear membrane and along the axon branches (arrows head) using an *in situ* hybridization assay; **C**) Viral growth curve showing a peak of BoHV-5 antigens and cytopathic effect detection at 72 h p.i.; **D**) Percentage of neuron-like cells positively labeled for viral DNA at all p.i. time points; **E**) Real-time PCR was used to quantify the mRNA levels and fold-changes were calculated by 2 -^ΔΔ^*Ct* method as compared with expression of endogenous bovine GAPDH mRNA level to normalization. The infected neuron-like cells revealed *glyco-C* mRNA synthesis at same levels from 24 h to 120 h post-infection (p < 0.05).

The susceptibility of neuron-like cells to BoHV-5 infection was investigated by assessing the viral growth curve, cell viability, and cytopathology. The one-step kinetic growth curve profile for infected neuron-like cells was established. CPE are an important parameter in the *in vitro* characterization of viral lytic potential*.* To study the CPE, an indirect measure of viral cell-to-cell spread – morphology, viral antigens, and DNA were monitored. Following infection, CPE was characterized by an enlargement of the neuronal branches (Figure 5A-D) and by appearance of small and large vesicles (Figure 5E-F). BoHV-5 antigens were detected by immunofluorescence at 96 h p.i. (Figure 6A) and active BoHV-5 replication was detected by ISH (Figure 6B). The viral DNA label was observed almost exclusively in the perinuclear region of infected neurons and ranged from sporadic to dense grain accumulations along neuron branches (Figure 6B). Almost 60% of neurons demonstrated active viral DNA replication by 72–96 h p.i. (500 cells per time-point in two parallel experiments) (Figure 6D). In the replication curve, maximum viral titre was observed at 72 h p.i. (Figure 6C) and loss of cell viability occurred only after 96 h p.i. (Figure 5G). Altogether, these results demonstrate that differentiated bWJ-MCS cells support productive BoHV-5 replication.

## Discussion

Several studies have described the ability of Wharton’s jelly cells from a variety of animal species to differentiate into neurons and/or glial cells *in vitro*[3]. The most commonly applied protocol to induce undifferentiated MSC cells to differentiate into neuron-like cells is based on treatment with bFGF (basic fibroblast growth factor), dimethylsulfoxide (DMSO) and butylatedhydroxyanisole (BHA) [7]. Some differences have also been described in the use of retinoic acid (RA) and/or insulin associated with hydrocortisone [26]. Retinoic acid is an oxidized derivative of retinol and is the active form of vitamin A. RA functions as a specific ligand for the retinoic acid receptor signaling pathway, which is required in embryonic development, bone formation, and in the maintenance of normal epithelial structures. The addition of RA to induction medium has been shown to influence neuronal differentiation and to improve cell survival [26].

The precise composition of the culture medium is critical to avoid inconsistent results, especially when the reagents are obtained from different sources. With regard to RA, most of the neural induction methods in rodent or human MSCs employ a combination of RA with other chemical agents, cytokines, and growth factors [7-9]. In the present study, a commercially defined medium with no supplement was used to facilitate the reproducibility of the culture system. Another advantage of the culture system developed herein is the natural adherence of neuron-like cells to the plastic flasks. This phenotype is different from neuron-like cells derived from fetal brain tissue which require a specific substrate to initiate differentiation [10]. Collectively, the results presented here indicate that it is possible to derive neuron-like cells from bWJ-MSCs. These differentiated cells maintain identical morphology and cell viability after 28 days of *in vitro* culture with a commercial medium. Moreover, there are no ethical issues in using cells *in vitro* from umbilical cords versus fetal brains. Following neuronal differentiation, the neuron-like cells clearly exhibited their neurogenic potential by expressing classic neuronal markers such N200, MAP2, NT3, Tau and GFAP. Nestin, CXCR4, and TuJI were also detected, but at lower intensities. Importantly, none of these neuronal markers were detectable inundifferentiated cells, suggesting the lack of spontaneous neuronal potential. However, a previous study using porcine `WJ-MSCs described similar expression levels of neuron-specific enolase (NSE) in differentiated and undifferentiated cells [9]. This finding has also been reported for bone marrow stromal cells isolated from rats, pigs, and humans [7,8]. Neurofilaments and GFAP, both considered important neuronal/glial proteins, were also found to be expressed in undifferentiated and differentiated MSCs using RA as a neuronal inducer for human bone marrow stromal cells [8]. Furthermore, flow cytometric analysis found that differentiated bWJ-MSCs expressed NT3, GFAP, MAP2, Tau, and N200. This indicates the presence of neural precursors, mature neurons, and glial cells in the same culture. Similar results have been reported with human neural progenitors obtained from umbilical cord blood; however, no GFAP expression could be detected in this experiment [7,8]. Thus, phenotyping of neuron-like cells revealed that more than 80% of the initial cell population became differentiated and expressed neurological receptors after 28 days of induction. This time period was inferior when compared to neural stem cells produced from fetal brain in humans [10], but similar to one study performed in dogs using Wharton’s jelly source [12].

The neurotropic RNA and DNA viruses that produce CNS diseases in humans and animals use a variety of pathological mechanisms. For members of the α- *Herpesvirinae* subfamily*,* almost all types and areas of the nervous system have been identified as targets for viral replication [16,18]. Although many studies have been performed *in vivo* using rabbits and cattle, it is difficult to examine BoHV-1 replication *in vivo.* an *in vitro* culture of rabbit sensory neurons and ganglionic non-neuron cells to examine BoHV-1 replication [27].

Because of this difficulty, suitable *in vitro* systems implementing the three R’s principle of Russel and Burch (1959) are needed to study primary host-virus interactions [27]. Because of the clinical relevance of the α-subfamily *Herpesvirinae*, *in vitro* models would be highly appreciated to replace animal studies. In addition to ethical considerations, *in vitro* studies would eliminate potential confounding factors such as individual animal variation and environmental factors [28].

Both herpes simplex virus 1 (HSV-1) and BoHV-1/5 establish lifelong latent infections in rabbit and cow sensory nerve ganglia [18,27]. Several days after infection by HSV-1 and BoHV-1/5, some neurons are productively infected (acute phase) [27]. Other neurons, however, become latently infected [18,23]. In the present study, the absence of floating neuron-like cells following BoHV-5 infection suggests a low rate of cell death. This was confirmed by MTT assay results showing high cell viability and 60% ISH positivity. These findings can be explained by the expression of a latency-associated region (LTR) gene that is involved in neuronal survival during HSV-1 infection *in vitro*[17]. In addition, *in vitro* replication does not always correlate with *in vivo* viral behavior. Thus, it is particularly important to note that data obtained from *in vitro* studies are not necessarily representative of viral host interactions occurring *in vivo*[27].

However, the molecular mechanisms by which productive and persistent viruses produce nervous system diseases are diverse and not completely understood. The results obtained in this investigation open the door to questions about neuronal death/survival mechanisms following BoHV-5 infection and replication.

## Conclusion

This report provides the first characterization of neuron-like cells derived from bWJ-MSCs. We have demonstrated that these cells are susceptible to BoHV-5 infection. These results open the door to replace animal models to study particular aspects of BoHV-5 biology and pathogenesis. Our *in vitro* model reduces the need for animal experimentation. The neuron-like cells developed herein can be used as a viable alternative to investigate several aspects of BoHV-5 biology including neurotropism, neurovirulence, and gene expression.

## Methods

### Bovine Wharton’s jelly-derived multipotent mesenchymal stromal cells (bWJ-MSCs)

The bWJ-MSCs were isolated and characterized as previously described [13] according to the Animal Care Committee at the University of São Paulo State, Brazil. The cell monolayers have been maintained in the laboratory of Animal Virology and Cell Culture since 2010 following standard procedures for seeding, freeze-thawing, and propagation. Passage 30 undifferentiated bWJ-MSCs were kept at 3.5 × 10^5^ cells/mL in 25-cm^2^ tissue culture flasks (Falcon, BD) at 38.5°C in 5% CO_2_ in a humidified incubator. The cells were seeded with Advanced-DMEM (Invitrogen®, Life Technologies™, Carlsbad, CA, USA) with the addition of 10% fetal bovine serum (FBS; Sigma-Aldrich®, St. Louis, MO, USA), 2 mM glutamine and 10 mM non-essential amino acids (Sigma-Aldrich®). The cells were then expanded until they reached subconfluence (80–90%) in serum-free conditions, at which point the supplement was switched to Xeno-Free™ (Invitrogen™) in lieu of FBS (Invitrogen™). The medium was refreshed every 24 h, and pictures were taken to observe the morphology of undifferentiated bWJ-MSCs.

### Differentiation of bWJ-MSCs

The differentiation protocol was divided into three stages. In stage I, bWJ-MSCs were resuspended at 2.8 × 10^4^ cells/mL in Neurobasal™ medium supplemented with 10% fetal bovine serum (FBS, Sigma-Aldrich®) for 24 h. Stage II consisted of replacing the culture medium with Neurobasal™ and B27 supplement (Invitrogen®). The medium was replaced after 24 h of induction; and stage III was started by replacing the old medium with Neurobasal™ with the addition of a neuro-differentiation supplement (Invitrogen®). Stage III was sustained for 15 consecutive days. The neuron-like cells were cultured both in 25-cm^2^flasks for the viability assay and on Lab-Tek® well chamber slides for immunostaining (Nunc™, Rochester, NY, USA). All of the media were supplemented with penicillin (100 IU/mL) and streptomycin (100 μg/mL). All cultures were incubated at 38.5°C in 5% CO_2_ with 95% humidity.

### Immunostaining for neural cell markers

Differentiated cells at 28 days of culture were fixed with 4% paraformaldehyde for 15 min in Lab-Tek® chamber slides (Nunc™). The cells were permeabilized for 10 min at room temperature in 0.4% Triton X-100 diluted in phosphate buffered solution (PBS). The fixed neuron-like cells were incubated overnight at 4°C with each of the primary antibodies anti-Nestin (cat # N5413, diluted at 1:100), anti-NT3 (neurotrophin 3; cat # SAB3300030, 1:50), anti-CXCR4 (chemokine receptor 4; cat # C3116, 1:20), anti-GFAP (cat # WH0002670M1, 1:50), anti-MAP2 (microtubule associated protein 2; cat # M1406, 1:20), anti-Tau (cat # T6402, 1:200), anti-ubiquitin (cat # GW10073F, 1:200), anti-β tubulin III (neuronal TuJI, cat # T8578, 1:100), and anti-N200 (neurofilament200k; cat # N4142, 1:200), all purchased from Sigma-Aldrich®. On the next day, after three washes, cells were incubated with the respective goat secondary antibody (1:100) anti-mouse, anti-rabbit and anti-chicken FITC (Sigma-Aldrich®). For nuclear staining, DAPI (1 mg/ml) was diluted in Fluormount™ aqueous medium and loaded onto samples for 15 min. The images were collected under an AxioImager® A.1 light and an ultraviolet (UV) microscope connected to an AxioCam®MRc (Carl Zeiss, Oberkochen, Germany). The images were processed using AxioVision® 4.8 software (Carl Zeiss) for each antigen, whereas values were determined by visually counting positive cells (−, 0%; +, 0-25%; ++, 25-50%; +++, 50-75%; ++++, 75-100%). The undifferentiated cells underwent the same staining procedure to assess for cross reactions among antibodies. The negative controls consisted of incubation of slides with bovine IgG and IgM isotypes as the primary antibodies.

### Flow cytometric analysis for neural cell markers

Following differentiation at 28 days of culture, 1 × 10^6^ differentiated cells were harvested after being detached with 0.25% trypsin (Sigma-Aldrich®), washed with PBS, permeabilized in 4% of paraformaldehyde, and incubated for 18 h at 4°C with the monoclonal antibodies at the same dilutions described for immunostaining in 1% Triton X-100 and 0.5% bovine serum albumin (BSA). After incubation with the primary antibodies, the cells were washed three times with PBS plus 0.1% Triton X-100. Next, a 1:50 dilution of the secondary antibody was added to 100 μl of the cell suspension and then incubated at 37°C for 30 min. The cell suspension was washed as previously described. After the final wash, the cells were fixed with 4% paraformaldehyde. Data were acquired with an Attune™ acoustic focusing cytometer system (Applied Biosystems™, Foster City, CA, USA) and at least 50,000 events were counted. The negative pattern was examined by applying the same cell suspension with the initial incubation with bovine IgG and IgM isotypes, and the result was included in the global compensation to exclude auto-fluorescence. A BL1-A (488 nm) filter was used in each analysis.

### Reverse transcription-polymerase chain reaction

Reverse transcription-polymerase chain reaction (RT-PCR) analysis was used to evaluate the differentiation of bWJ-MSCs into neuron-like cells. Briefly, total RNA was extracted from the cell culture at 28 days using the TRizol™ method (Invitrogen®). mRNA expression of the nestin, N200, β-tubulin III (TuJI), GFAP and NT3 genes was reverse transcribed using an enhanced avian RT first strand synthesis kit (STR-1, Sigma-Aldrich). The PCR reaction was conducted following JumpStart™ Taq ready mix according to manufacturer’s instructions (Sigma-Aldrich®) with the specific primers described in Table 2. A housekeeping gene, H2A (bovine histone), was used as an internal control. All of the primers were designed to amplify 207 to 209 bp and were electrophoresed on a 1.5% agarose gel. Samples were visualized by ethidium bromide staining and photographed.

**Table 2:** Cell surface neurogenic markers analysed by RT-PCR

**Genes**	**Accession number**	**Primers sequence**	**Size (bp)**
Nestin	AB257750	forward 5`- AGACTTCCCTCAGCTTTCAGG-3`	209
		reverse 5`-GCCTGGAGGAATTCTTGGTT-3`	
N200	NM174121	forward 5`TAGCACATTTGCAGGAAGCA-3`	208
		reverse 5`-CGGCCAATTCCTCTGTAATG-3`	
β-tubulin III	NM001077127	forward 5`- AGATTCCGCTATTAGCTA-3`	207
		reverse 5`- CGGTACCGGTTAATAGTAGT-3	
GFAP	L19867	forward 5`- TGCAGACCTGACAGACGCTGTTG-3`	209
		reverse 5`-CTGCTAGAGGGCGAGGAGAACG-3`	
NT3	NM001077988	forward 5`- ATGCCGTAGCGGTTAATGCA-3`	208
		forward 5`- ATGCCGTAGCGGTTAATGCA-3`	
H2A	AW461431	forward 3`-GTCTTGGAGTACCTGACCGC-5`	209
		reverse 3`-AGTCTTCTTCGGGAGCAACA-5`	

### Susceptibility of neuron-like cells to viral infection

After differentiation, neuron-like cells were maintained in a Neurobasal™ medium (Invitrogen™) plus B27 without FBS. The BoHV-5 strain used in the experiment was isolated from an outbreak of meningoencephalitis [22]. This strain has 90% sequence similarity to EVI99 based on the US9 region (GenBank accession number AY064172). The strain was kept for 9 consecutive cell passages in our laboratory [29]. For the infection, neuron-like cells were grown to 70% confluence in Lab-Tek® chambers slides (Nunc™) and infected with 100 μl of a BoHV-5 suspension (10^3.2^ TCID_50_/ml), corresponding to a multiplicity of infection (m.o.i.) of 0.1. The viral suspension was allowed to adsorb for 1 h at 38.5°C. After incubation, the viral suspension was removed and the monolayers were supplemented with the same medium. Inoculated cells were monitored thereafter for cytopathic effect (CPE).

### Viability assay and viral growth kinetics

The cell proliferation analysis was performed using an In Vitro Toxicology Assay® Kit, an MTT-based assay, in both infected and non-infected neuron-like cells at 0, 24, 48, 72, 96 and 120 h post-infection (p.i.) (TOXI-1 Kit; Sigma-Aldrich®). For each p.i. time point, the culture supernatant was removed and 2 mL of MTT (tetrazolium salts) were added following the manufacturer’s recommendations (Sigma-Aldrich®). Absorbance was measured at 600 nm with a Biophotometer (Eppendorf®, Hamburg, Germany). All reported values are means of triplicated samples.

One-step growth curve of the BoHV-5 in neuron-like cells was assayed in a multiplication kinetics experiment, followed by infection at 80% of monolayer confluence with m.o.i of 1. After 90 min of adsorption at 38.5°C, the inoculum was removed and fresh medium was added. The neuron-like cells were incubated at different time intervals (24, 48, 72, 96 and 120 h p.i). After incubation, both supernatant and lysate cells were harvested and assayed for the presence of the virus by infecting (Madin-Darby bovine kidney cells) MDBK cells as described previously [30]. All experiments were performed in triplicate. Infectious virus titres were calculated according to the Spearmann-Kärber method and expressed as Log_10_ TCID_50_/mL.

### Viral nucleic acid detection by in situ hybridization assay

To estimate the number of neurons actively replicating viral DNA, neuron-like cells grown on Lab-Tek® (Nunc™) chamber slides were infected with at least 1 TCID_50_ per cell, incubated for 24, 48, 72, 96 and 120 h p.i. and fixed with 95% ethanol-glacial acetic acid (3:1) for 30 min in an ice bath. *In situ* hybridization assay (ISH) was performed as described previously [21,31,32]. To perform *in situ* hybridization, the DNA probe was prepared from PCR amplicons of BoHV-5 DNA glycoprotein-C gene according to previous study [28,29,32]. The 159 base pair (bp) amplicons were produced by primary forward PCR primer gI + (5`- GTG CTC TTC TCC ATC GCC-3`) and reverse primer gI- (5`-GCG GAG GAGGAG TTG TCG G-3-bio`) (Invitrogen™, Brazil). After DNA purification from agarose gel, the PCR product was linked into the TA-vector (pGEMTEasy, Promega, Madison, WI, USA) and ligation products were introduced into *E. coli* by heat shock. Positive colonies were confirmed by DNA sequencing. A confirmed positive colony was cultured and plasmid DNA was prepared using a commercially available kit (Promega Mini- Prep®, Promega, Madison, WI, USA). The probe was generated by PCR reaction [31] targeting the gC gene located in the plasmid as described before using the biotin labeled reverse primer (Invitrogen™).

Briefly, for ISH assay the infected and uninfected neuron-like cells were placed on glass slides (EasyPath®), pre-treated with pure poly-L-lysine (Sigma-Aldrich®) and fixed with 4% (w/v) paraformaldehyde (Sigma-Aldrich®) in phosphate-buffered saline (PBS) for 24 h at 4°C. Slides were treated with proteinase *K* (10 μg/mL, Invitrogen™) for 10 min at room temperature and washed in PBS. A 159-bp denatured biotin labeled probe consisting of 2 μL (2 ng/mL) of probe and 98 μL of pre-hybridization buffer (50% formamide, 5% bovine seroalbumin, 1% N-lauroylsarcosine and 0.02% sodium dodecyl sulphate, Sigma-Aldrich®) was applied the slides. The slides were incubated overnight at 37°C under a plastic coverslip in a humidified chamber. The slides were then washed and excess probe was removed by washing in increasingly stringent solutions consisting of 1x SSC (saline sodium citrate) and 0.1x SSC for 10 min at 42°C. The detection system consisted of incubation with monoclonal antibody against biotin labeled to FITC (Sigma-Aldrich®).

The ISH positive reaction was visualized after the slides were covered using Fluormount® (Sigma-Aldrich®) medium and observed under a UV microscope. The images were collected under an Axio Imager A.1 light microscope connected to an AxioCamMRc (Carl Zeiss Oberkochen, Germany), and the micrographs were processed with AxioVision 4.7 software (Carl Zeiss). The results were expressed as the mean percentage of neurons positively marked.

### Total RNA isolation and quantitative real time polymerase chain reaction (qPCR)

Upon harvesting at 24, 48, 72, 96 and 120 h p.i., the cells, monolayers and respective supernatants, and total RNA was extracted using the Trizol LS™ protocol according to manufacturer’s instructions (Invitrogen®). An average of 150 ng of total RNA was used for first-strand cDNA synthesis with Enhanced Avian RT First Strand Synthesis (Sigma-Aldrich®). The qPCR was carried out and analyzed by the software on a StepOnePlus® real time instrument (Applied Biosystems™). The real time PCR mixtures (50 μl) contained 1.2 μg of cDNA, 400 nM primers and 200 nM probes. The probes were FAM-label customized for glyco-C gene sequences according to a previous report [33]. The PCR was initiated by sequential amplification of 40 cycles at 95°C (15 s) and 60°C (60 s). The results were obtained from three replicates of each sample to ensure representative and accuracy pipetting. The expression of bovine GAPDH (glyceraldehyde-3-phosphate dehydrogenase) gene was also quantified in a similar way for normalization. The comparative delta-delta C_*t*_ method was used to analyze the results. The expression level of the BoHV-5 *glyco-C* gene at the corresponding time point in infected and uninfected neuron-like cells was quantified in comparison to GAPDH C_*t*_ values.

### Statistical analysis

All of the experiments were performed in triplicate or greater. Results of representative experiments are presented. Descriptive statistics include the mean ± standard deviation (s.d.). Statistical analysis was performed using ANOVA to evaluate the results. Student’s *t*-test was used for comparison of uninfected (control) and BoHV-5 infected cells. Two-tailed p values < 0.05 were considered significant.

## Competing interests

The authors declare that they have no competing interest.

## Authors’ contributions

TCC, JBN and TFA designed and directed studies, and were involved in the interpretation of the data. MCF, CSF and HFF participated in immunofluorescence assay. RG and HFF performed cytometry analysis. EFF helped in the manuscript design and discussion of results. All authors read and approved the final manuscript.
